# Immune and cytokine alterations and RNA-sequencing analysis in gestational tissues from pregnant women after recovery from COVID-19

**DOI:** 10.1186/s12879-023-08607-z

**Published:** 2023-09-21

**Authors:** Xing Xin, Weiqi Yao, Zijing Zhang, Xin Yang, Shufang Li, Ying Zhu, Cong Zhang, Long Zhang, Hailong Huang, Tengyun Dong, Haibo Dong, Ling Feng, Shaoshuai Wang

**Affiliations:** 1 Department of Obstetrics and Gynecology, Tongji Hospital, Tongji Medical College, Huazhong University of Science and Technology, No. 1095 Jiefang Road, Wuhan, 430032 Hubei P.R. China; 2 Wuhan Optics Valley Vcanbiopharma Co. Ltd, Wuhan, 430000 Hubei P.R. China; 3 VCANBIO Cell & Gene Engineering Corp., Ltd, Tianjin, 300384 P.R. China; 4 Department of Biology and medicine, Hubei University of Technology, Wuhan, 430068 Hubei P.R. China; 5 Wuhan Optics Valley Vcanbio Cell & Gene Technology Co., Ltd, Wuhan, 430000 Hubei P.R. China; 6 Hubei Engineering Research Center for Human Stem Cell Preparation, Application and Resource Preservation, Wuhan, 430000 Hubei P.R. China; 7 Department of Medical office, Wuchang Shouyi College Hospital, Wuhan, 430064 Hubei P.R. China; 8 Department of Rehabilitation Medicine, Zhongnan Hospital of Wuhan University, Wuhan, 430077 Hubei P.R. China

**Keywords:** COVID-19, Pregnant women, Human umbilical cord mesenchymal stem cells, Human amniotic mesenchymal stem cells, Cytokines, Lymphocyte subsets

## Abstract

**Background:**

COVID-19 is a global pandemic. Understanding the immune responses in pregnant women recovering from COVID-19 may suggest new therapeutic approaches.

**Methods:**

We performed a cross-sectional study between March 1, 2020, and September 1, 2020. Participants were assigned into the convalescent COVID-19 group if they had a previous COVID-19 infection during pregnancy or the healthy control group. RNA-Seq was performed on human umbilical cord mesenchymal stem cells (hUMSCs) and human amniotic mesenchymal stem cells (hAMSCs). Immunohistochemical staining, cytokine testing, lymphocyte subset analysis, RNA-Seq, and functional analyses were performed on the placental and umbilical cord blood (UCB) and compared between the two groups.

**Results:**

A total of 40 pregnant women were enrolled, with 13 in the convalescent group and 27 in the control group. There were 1024, 46, and 32 differentially expressed genes (DEGs) identified in the placental tissue, hUMSCs, and hAMSCs between the convalescent and control groups, respectively. Enrichment analysis showed those DEGs were associated with immune homeostasis, antiviral activity, cell proliferation, and tissue repair. Levels of IL-6, TNF-α, total lymphocyte counts, B lymphocytes, Tregs percentages, and IFN-γ expressing CD4^+^ and CD8^+^ T cells were statistically different between two groups (*p* ≤ 0.05). ACE2 and TMPRSS2 expressed on the placenta were not different between the two groups (*p* > 0.05).

**Conclusion:**

Multiple changes in immune responses occurred in the placental tissue, hUMSCs, and hAMSCs after maternal recovery from COVID-19, which might imply their protective roles against COVID-19 infection.

**Supplementary Information:**

The online version contains supplementary material available at 10.1186/s12879-023-08607-z.

## Background

Coronavirus disease 2019 (COVID-19) is a global pandemic [1, 2]. During SARS-CoV-2 infection, angiotensin converting enzyme-2 (ACE2) receptor and transmembrane serine protease 2 (TMPRSS2) are responsible for allowing SARS-CoV-2 to enter cells [3]. Once infection occurs, the host immune responses can provide antiviral protection or may contribute to the pathogenesis and severity of the virus. The early response involves immune suppression and tight junction dysregulation, which may provide a defense against the infection [4–6]. However, the late response could lead to a cytokine storm, which can subsequently lead to multiple organ injuries and even death [4]. In addition, lymphopenia, where T and B cell numbers are reduced could be associated with poor memory B cell antibody responses [6, 7]. Naive T-cell responses are related to the subsequent uncontrollable cytokine production and the severity of the COVID-19 infection. After recovery from acute COVID-19, some convalescent COVID-19 patients may still have various complications and sequelae, such as pulmonary fibrosis, digestive disorders, and spermatogenesis dysfunction, suggesting persistent inflammation in these patients. Therefore, further investigation of the immune responses after the SARS-CoV-2 infection could not only clarify the pathogenesis of COVID-19, but also provide clues for novel treatments.

The majority of neonates born to COVID-19 pregnant women were not infected with the virus [8–12]. It was reported that the immune cells in the decidua basalis and the maternal placental component could play an important role in anti-viral responses during the pregnancy [13]. In addition, mesenchymal stem cells, which are multipotent cells with immune activities that can be found in the umbilical cord or amniotic fluid, are known to have immunomodulatory, therapeutic, and tissue repair properties. Several clinical trials had demonstrated that umbilical cord mesenchymal stem cells (hUMSCs) and human amniotic mesenchymal stem cells (hAMSCs) were effective and safe in the treatment of severe COVID-19 infection [14–18]. Some researchers have proposed that MSCs and derived extracellular vesicles could be a promising therapeutic strategy for post-COVID-19 complications [19, 20]. More studies are required to delineate the inflammatory responses in pregnant women.

In the present study, we were interested in finding the differences in hUMSCs and hAMSCs between pregnant women recovered from COVID-19 and healthy pregnant women. We also compared differentially expressed genes (DEGs), cytokine levels, lymphocyte subsets, and cellular functions, as well as ACE2 and TMPRSS2 expression, in placental tissues between the two groups. We further discussed the potential theoretical basis for choosing MSCs to treat or prevent COVID-19 and COVID-19-associated diseases.

## Methods

### Study design and participant selections

We performed a cross-sectional study at the Tongji Hospital, Wuhan, China, between March 1, 2020, and September 1, 2020. The study protocol was approved by the ethics committee of Tongji Medical College, Huazhong University of Science and Technology (No. 2020S129, 2020S176). All study participants provided written informed consent.

Pregnant women who had COVID-19 during pregnancy and then recovered were enrolled in the convalescent group. SARS-CoV-2 nucleic acid tests were performed in samples from nasal swabs in the convalescent group. Two genes of Nucleocapsid protein (N) and Open Reading Frame1ab (ORF1ab) were amplified and targeted by real-time polymerase chain reaction (RT-PCR) (Maccura i3000 kit, Chengdu, China) with primers listed in Table 1 [21]. COVID-19 was diagnosed using a positive SARS-CoV-2 nucleic acid test. Recovery from COVID-19 was defined as two negative SARS-CoV-2 nucleic acid tests taken 24 h apart [22]. Pregnant women with negative SARS-CoV-2 nucleic acid, IgG, and IgM tests were enrolled in the control group. Pregnant women in the convalescent group who tested positive with the SARS-CoV-2 nucleic acid test were excluded from the study.

**Table 1:** Primers for the real-time polymerase chain reaction

Gene	Forward primer	Reverse primer	Probe
Target N	GGGGAACTTCTCCTGCTAGAAT	CAGACATTTTGCTCTCAAGCTG	5’-FAM-TTGCTGCTGCTTGACAGATT-TAMRA-3’
Target ORF1ab	CCCTGTGGGTTTTACACTTAA	ACGATTGTGCATCAGCTGA	VIC-CCGTCTGCGGTATGTGGAAAGGTTATGG-BHQ1-3’

### SARS-CoV-2 nucleic acid and IgM/IgG antibody tests

Nucleic acid and IgM/IgG antibody tests for SARS-CoV-2 were performed in the hospital laboratory. The SARS-CoV-2 IgM/IgG antibody detection kit was purchased from Maccura Biotechnology (Chengdu, China). The presence of SARS-CoV-2 in respiratory specimens, maternal peripheral blood, and umbilical cord blood (UCB) samples were detected using RT-PCR.

### Cytokine detection and lymphocyte immunophenotyping

Newborn UCB was obtained immediately after delivery. Serum biochemical tests were carried out in the hospital laboratory. Interleukin (IL)-1β, IL-2R, IL-8, IL-10, and tumor necrosis factor (TNF)-α were detected using the Siemens chemiluminescent method (Siemens, Germany) [23]. IL-6 was detected using the Roche electrochemiluminescence method (Roche, Switzerland) [23]. Lymphocyte subset immunophenotyping and functional analyses were carried out using a BD flow cytometer (BD Biosciences, USA) [23].

### Immunohistochemical staining of placental tissue

The placenta was obtained immediately after the delivery. Samples of villous tissue were taken from two separate areas in the placental midzone. These included the full thickness of the placenta, extending from the fetal to the maternal surface, including both amnion and decidua [24]. The tissue was quickly fixed in 4% formalin and then embedded in paraffin. Immunohistochemical (IHC) staining was performed on selected slides using antibodies for ACE2 (Abcam, AB108252, 1:200, UK) and TMPRSS2 (Abcam, AB109131, 1:200, UK). Sections were then visualized using a Panoramic Scanner (Pannoramic DESK, P-MIDI, P250, Hungary) with Caseviewer C.V 2.3 used for histopathological diagnosis. Six study researchers (XX, XY, SL, YZ, CZ, and LZ) independently scored the stained slides. The results were averaged to obtain a final score. IHC scoring rules are detailed in the supplementary material IHC score rules.

### Isolation of hUMSCs and hAMSCs

A 0.5 cm section of umbilical cord without blood vessels was obtained from each neonate and placed in a sterile centrifuge tube with 0.25% trypsin (Gibco, USA). Human amnion membrane (hAM) tissue was cut into sections and placed into a sterile centrifuge tube containing 0.25% trypsin. Both samples were immersed in phosphate buffered saline (PBS) with 0.75 mg/ml of collagenase type IV (Sigma, USA) and 0.075 mg/ml DNase I (Takara, Japan). Pellets were obtained using a cell strainer, suspended in DMEM/F12 (1:1) media (Gibco, USA) with 10% fetal calf serum (Gibco, USA) and then cultured. Complete medium was replaced every three days. Cells were passaged when they reached confluence. In this study, we used third passage cells for subsequent experiments.

### Phenotypic analysis of hUMSCs and hAMSCs

The phenotype of cultured hUMSCs and hAMSCs was assessed with flow cytometry using a human mesenchymal stem cell detection kit. The kit included mixed CD73-FITC/CD105-PE, CD45-FITC/HLA-DR-PE, and IgG1-FITC/IgG2-PE antibodies (Quantobio Co., Ltd., China) and was performed according to the manufacturer’s instructions. Briefly, cells were centrifuged at 300 g for 5 min. After removing the supernatant, washing with PBS, discarding the supernatant, and re-suspended cells to 5 × 10^6^-6 × 10^6^/mL. Each testing tube contained approximately 100 cells/µL. CD73-FITC/CD105-PE, CD45-FITC/HLA-DR-PE, and IgG1-FITC/IgG2-PE antibodies (5 µL) were added and mix thoroughly. The solution was incubated at the room temperature and shielded from light for 15 min. After washing with PBS, centrifugation, re-suspension, the cells were stained and analyzed in a LSR II flow cytometer. DIVA (BD Biosciences, USA) and FlowJo software were used to analyze cell phenotypes.

### RNA-Sequencing analysis

Decidual tissue samples were isolated from the maternal-facing surface of the placenta. The dissected tissues were cut into small pieces, immersed into 1ml TRIzol® reagent (Invitrogen, USA) immediately, and stored at -80 °C. Total RNA was extracted from hUMSCs, and hAMSCs using a RNEASY Midi kit (Qiagen, USA), according to the manufacturer’s instructions (Wuhan Metware Co., Ltd. and Beijing Mygenostics Co., Ltd., China). A Qubit 2.0 spectrophotometer and Aligent 2100 Bioanalyzer were used to quantify and evaluate the quality and purity of the isolated RNA. mRNA was enriched using the Oligo (dT) and cDNA synthesis primed by random hexamer. RNA-Sequencing analysis was performed in the Wuhan Metware Co., Ltd. and Beijing Mygenostics Co., Ltd. Laboratories in China. Ribo-depleted libraries were prepared and samples were sequenced on an Illumina HiSeq. Fragments per kilobase million (FPKM) expression values of the genes reflected changes in the gene expression profile after SARS-CoV-2 infection. The raw reads were aligned to the human reference genomes hg38. To test intracellular virus, open reading frames of SARS-CoV-2 (ORF1ab, nsp1, nsp2, nsp3, nsp4, nsp5, nsp6, nsp7, nsp8, nsp9, nsp10, RdRp, nsp13, nsp14, nsp15, nsp16, ORF1ab, nsp11, S, ORF3a, E, M, ORF6, ORF7a, ORF7b, ORF8, N, and ORF10) were added to the reference genome before the alignment with BWA (NC_045512(https://www.ncbi.nlm.nih.gov/datasets/taxonomy/2697049/). The FC value of each gene was calculated using the DESeq2 software. FDR ≤ 0.05 and |Log2FC| ≥ 1 were used as the thresholds to identify DEGs between convalescent and healthy groups. Cutadapt, STAR, DESeq2, BWA, and VirusFinder softwares were used in this study for bioinformatics analyses (https://cutadapt.readthedocs.io/en/stable/; https://github.com/alexdobin/STAR; https://bioconductor.org/packages/release/bioc/html/DESeq2.html; https://bio-bwa.sourceforge.net/; https://bioinfo.uth.edu/VirusFinder/). The database of the Kyoto encyclopedia of genes and genomes (KEGG) was then used to analyze the upregulated or downregulated genes to identify possible cell signaling pathways [25–27].

### Statistical analyses

Continuous data are presented as mean ± standard deviation or median with interquartile ranges, depending on the data distribution. Categorical data are presented as numbers or percentages. An unpaired 2-sided Student’s test was used to compare the differences in cytokine levels and lymphocyte subpopulations between the convalescent and healthy groups. A *p* value of < 0.05 was considered statistically significant. All analyses were performed in SPSS for Windows version 20.0 (IBM Corp., Armonk, NY, USA).

## Results

### Baseline characteristics of study participants

A total of 40 pregnant women were enrolled into the study, with 13 in the convalescent group and 27 in the control group. During COVID-19 infection in the 13 convalescent women, one had a temporary loss of taste, one had occasional palpitations, and three had sleep disorder and fatigue. All of them recovered with no remaining clinical symptoms. The comparisons of baseline characteristics between the two groups are shown in Table 2. Other clinical characteristics of study participants are shown in the supplemental material metadata.

**Table 2:** Comparison between clinical and laboratory characteristics between convalescent and control groups

Characteristics	Convalescent group (N = 13)	Control group (N = 27)	*p*
Age	33.4 ± 4.4	34.8 ± 6.0	0.451
Gravida	2.5 ± 1.1	3.1 ± 1.9	0.359
Parity	1.8 ± 0.6	1.7 ± 0.7	0.779
Pregnancy outcomes			
Gestational age at delivery	256.2 ± 28.4	258.6 ± 17.7	0.786
Delivery mode			0.004
Vaginal delivery	7(53.8%)	2(7.4%)	
Cesarean section	6(46.2%)	25(92.6%)	
Complications	7(53.8%)	16(59.3%)	0.746
Neonate Apgar score			
1-minute	7.7 ± 0.9	7.8 ± 0.6	0.725
5-minute	8.8 ± 0.8	8.7 ± 0.9	0.822
Laboratory tests			
White blood cell count	9.58 ± 1.75	8.59 ± 2.87	0.258
Lymphocyte count	1.48 ± 0.4	1.51 ± 0.41	0.842
Red blood cell count	3.94 ± 0.37	3.94 ± 0.31	0.987
Platelet	185.4 ± 36.9	187.6 ± 47.6	0.886

### RNA-sequencing analysis of placental tissue

**Fig. 1:**
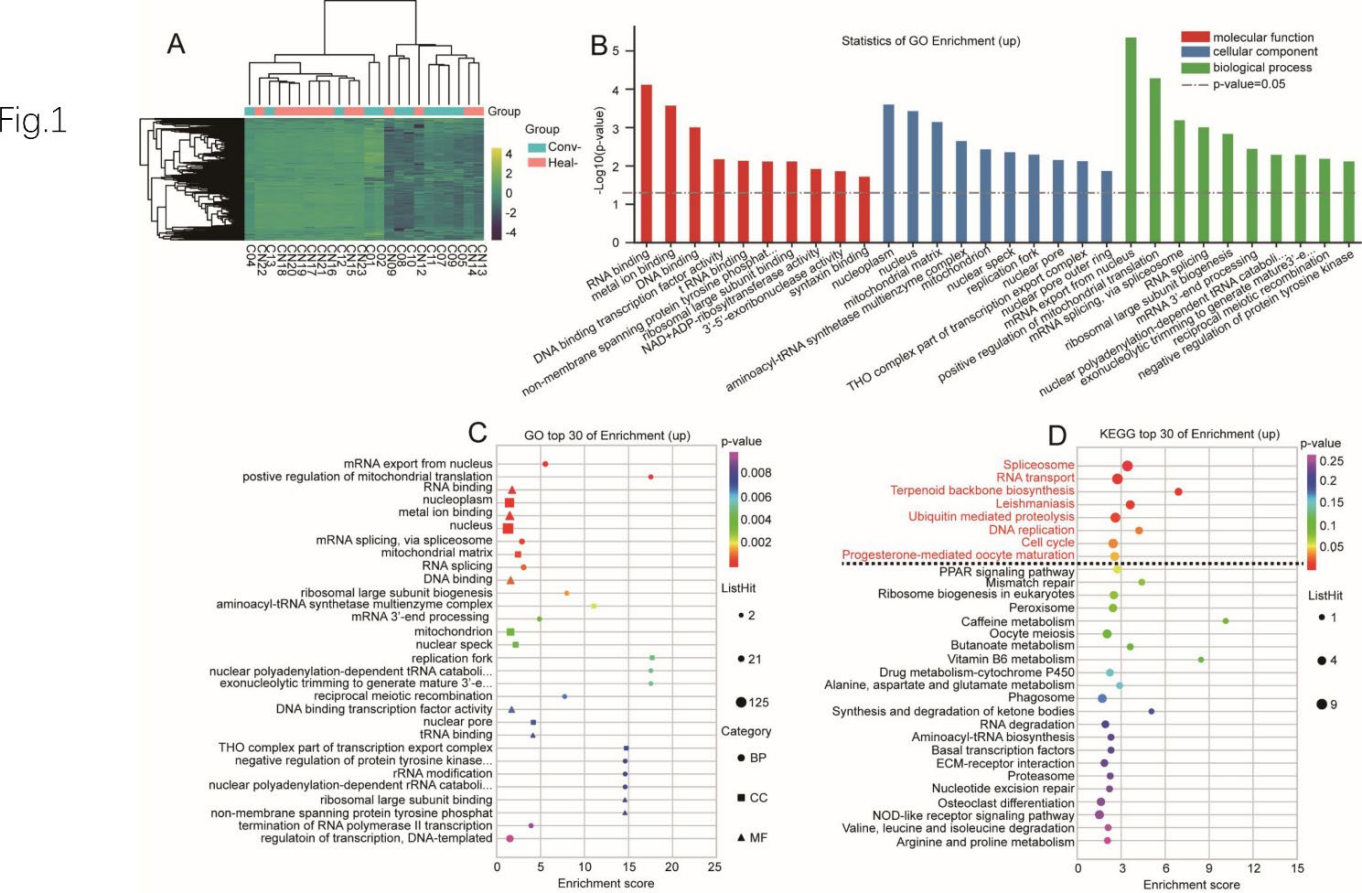
Differential expression of upregulated genes in decidua basalis between convalescent and control groups. **A:** Heatmap. Yellow and black stripes represent high and low gene expression, respectively. **B:** GO enrichment analysis. **C:** GO bubble chart. The vertical axis and horizontal axis represent the GO terms and the enrichment score, respectively. **D:** KEGG bubble chart. The vertical axis and horizontal axis represent enrichment pathway and enrichment score, respectively

RNA-sequencing analysis was performed on the decidua basalis from the convalescent group and healthy control group. Differential expression analysis identified 1,024 DEGs, including 614 upregulated genes and 410 downregulated genes between the two groups. Enrichment analysis of DEGs used the oebiotech online analysis tools (https://cloud.oebiotech.cn/task/). Upregulated genes were used to perform gene ontology (GO) enrichment and showed GO terms related to positive regulation of mitochondrial translation (*p* = 5 × 10^− 5^), negative regulation of protein tyrosine kinase activity (*p* = 0.0073), viral mRNA export from the host cell nucleus (*p* = 0.01), intracellular transport of virus (*p* = 0.016), G1/S transition of mitotic cell cycle (*p* = 0.02), positive regulation of interferon-alpha production (*p* = 0.021), myeloid cell differentiation (*p* = 0.025), viral processing (*p* = 0.027), cellular response to cytokine stimulus (*p* = 0.028), and T cell differentiation (*p* = 0.039). KEGG pathway enrichment analysis of upregulated genes identified 14 pathways, of which 8 KEGG pathways were significantly enriched (Fig. 1).

The other GO enrichment terms, KEGG pathways, upregulated placental tissue genes are included in the supplementary material 1 of sheet GO 1, sheet KEGG 1, and sheet 3.

**Fig. 2:**
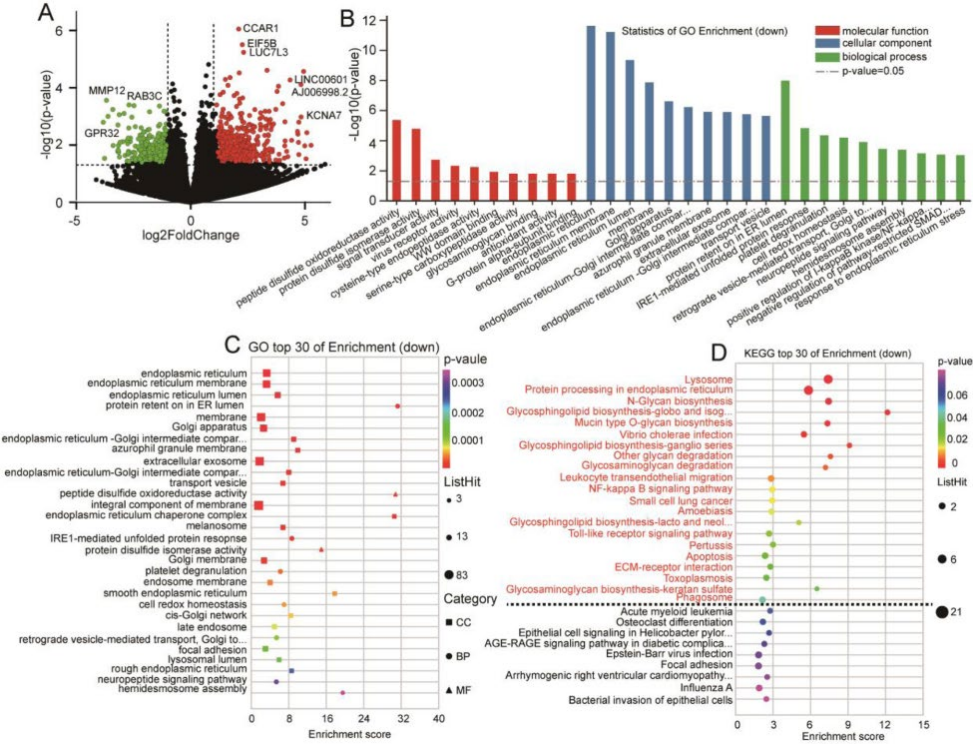
Differential expression of downregulated genes in decidua basalis between convalescent and control groups. **A:** Volcano plot. Red and green dots represent the upregulated and downregulated expressed genes expression, respectively. **B:** GO enrichment analysis. **C:** GO bubble chart. The vertical axis and horizontal axis represent the GO terms and the enrichment score, respectively. **D:** KEGG bubble chart. The vertical axis and horizontal axis represent enrichment pathway and enrichment score, respectively

Downregulated genes were used to performed GO enrichment and showed GO terms related to platelet degranulation (*p* = 4.1 × 10^− 5^), positive regulation of I-kappaB kinase/NF-kappa B signaling (*p* = 0.00063), virus receptor activity (*p* = 0.0043), extracellular matrix organization (*p* = 0.0045), intrinsic apoptotic signaling pathway in response to DNA damage by p53 class mediator (*p* = 0.0046), establishment or maintenance of cell polarity (*p* = 0.0063), neutrophil degranulation (*p* = 0.0077), negative regulation of cell migration (*p* = 0.0087), platelet dense granule lumen (*p* = 0.012), negative regulation of cell growth (*p* = 0.013), regulation of fibroblast migration (*p* = 0.015), regulation of cell migration (*p* = 0.019), cadherin binding involved in cell-cell adhesion (*p* = 0.02), cell-cell adherens junction (*p* = 0.022), angiogenesis (*p* = 0.026), wound healing (*p* = 0.027), heparin binding (*p* = 0.032), blood vessel remodeling (*p* = 0.033), negative regulation of T cell proliferation (*p* = 0.041), and B cell activation (*p* = 0.041). KEGG pathway enrichment analysis of downregulated genes identified 38 pathways and of these, 21 KEGG pathways were significantly enriched (Fig. 2). The other GO enrichment terms, KEGG pathways, and downregulated placental tissue genes are included in the supplementary material 2 of sheet GO 2, sheet KEGG 2, and sheet 3.

### hUMSCs RNA-seq analysis

**Fig. 3:**
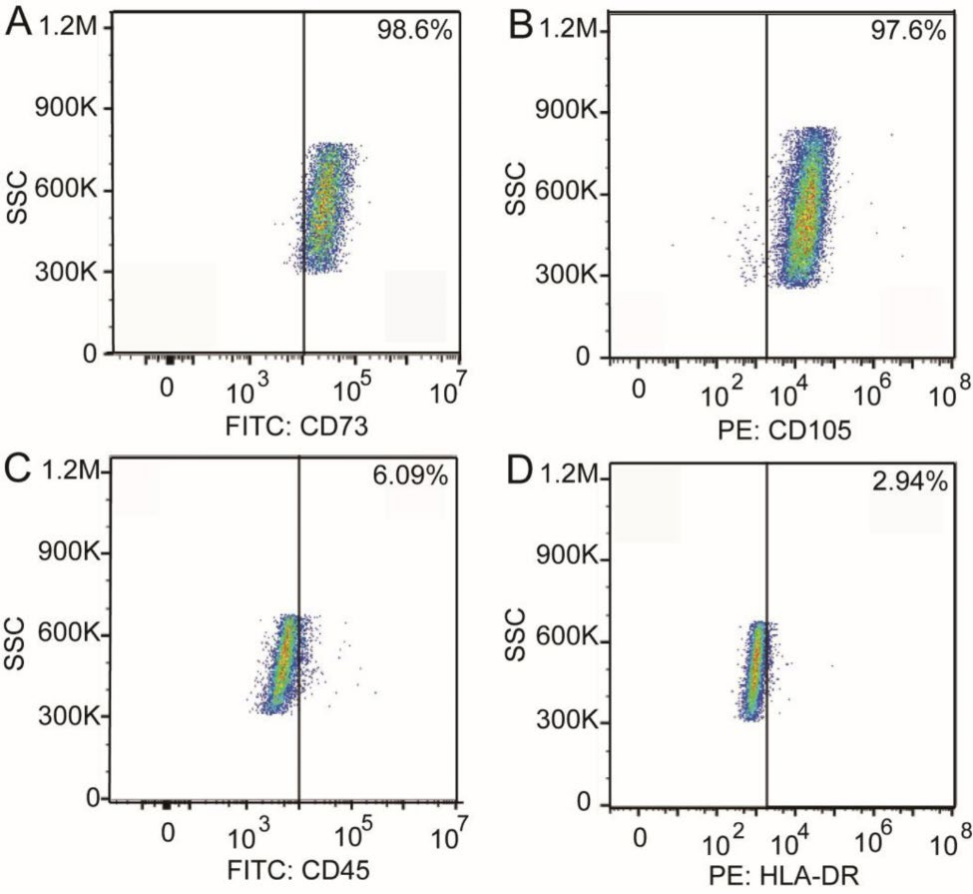
Cellular phenotype of hUMSCs as detected by flow cytometry. Cells are positive for CD73 and CD105 and negative for CD45 and HLA-DR.

**Fig. 4:**
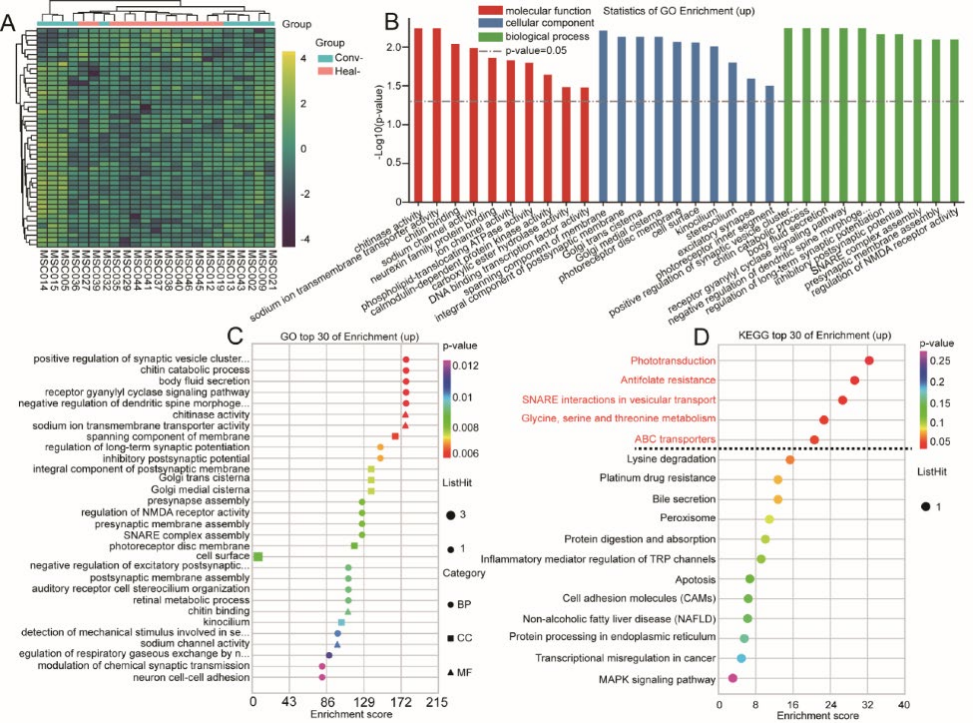
Differential expression of upregulated genes in hUMSCs between convalescent and control groups. **A:** Heatmap. Yellow and black stripes represent high and low gene expression, respectively. **B:** GO enrichment analysis. **C:** GO bubble chart. The vertical axis and horizontal axis represent the GO terms and the enrichment score, respectively. **D:** KEGG bubble chart. The vertical axis and horizontal axis represent enrichment pathway and enrichment score, respectively

The phenotype of hUMSCs was identified using flow cytometry. The expression of different phenotypic markers is shown in Fig. 3. Microscopy images of hUMSCs are shown in the supplemental material Figure S1. RNA-seq analysis was performed on hUMSCs from convalescent and healthy pregnant women. A total of 46 DEGs were identified, of which 39 were upregulated genes and 7 were downregulated genes. Upregulated genes were used to perform GO enrichment and showed GO terms related to positive regulation of synaptic vesicle clustering (*p* = 0.0056), receptor guanylyl cyclase signaling pathways (*p* = 0.0056), regulation of NMDA receptor activity (*p* = 0.0079), regulation of respiratory gaseous exchange by neurological system processes (*p* = 0.011), calmodulin-dependent protein kinase activity (*p* = 0.022), and cell adhesion molecule binding (*p* = 0.041). KEGG pathway enrichment analysis of upregulated genes identified 17 pathways, of which 5 KEGG pathways were significantly enriched (Fig. 4). The other GO enrichment terms, KEGG pathways, and upregulated hUMSC genes are included in the supplementary material 3 of sheet GO 3, sheet KEGG 3, and sheet 3.

**Fig. 5:**
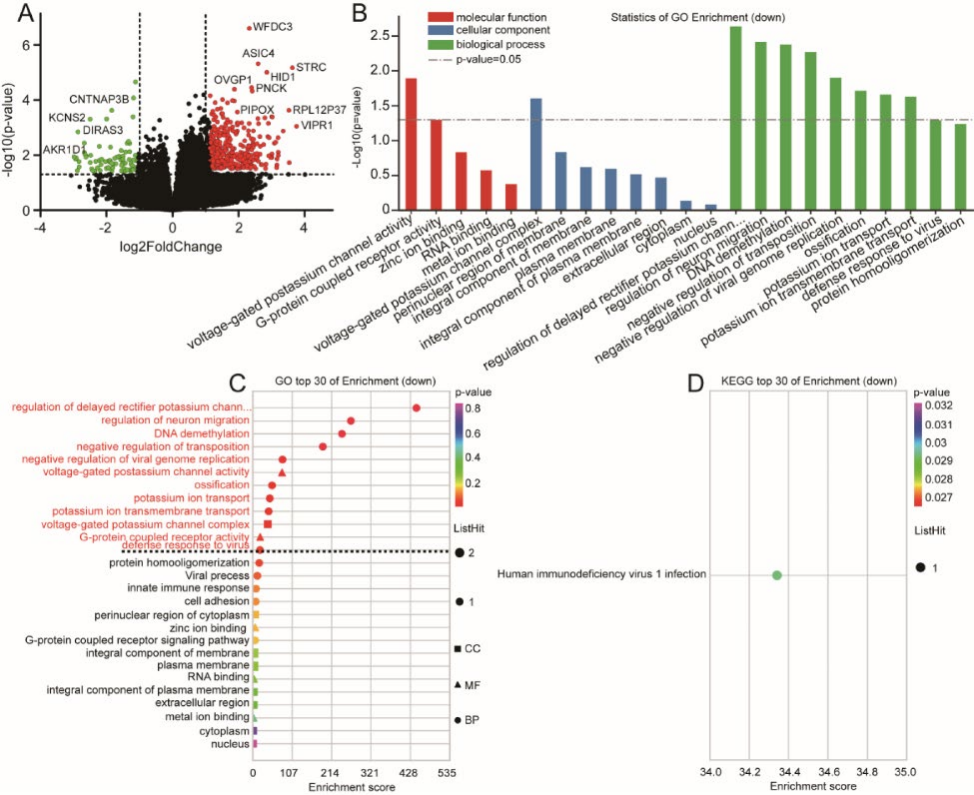
Differential expression of downregulated genes in hUMSCs between convalescent and control groups. **A:** Volcano plot. Red and green dots represent the upregulated and downregulated expressed genes, respectively. **B:** GO enrichment analysis. **C:** GO bubble chart. The vertical axis and horizontal axis represent the GO terms and enrichment score, respectively. **D:** KEGG bubble chart. The vertical axis and horizontal axis represent enrichment pathway and enrichment score, respectively

Downregulated genes were used to perform GO enrichment and showed GO terms related to regulation of delayed rectifier potassium channel activity (*p* = 0.0022), regulation of neuron migration (*p* = 0.0037), DNA demethylation (*p* = 0.0041), negative regulation of viral genome replication (*p* = 0.012), voltage-gated potassium channel activity (*p* = 0.013), ossification (*p* = 0.019), G-protein coupled receptor activity (*p* = 0.049), and host defense responses to virus (*p* = 0.05). KEGG pathway enrichment analysis of downregulated genes identified only one pathway, of which human immunodeficiency virus 1 infection (path: hsa05170, *p* = 0.029) was significantly enriched (Fig. 5). The other GO enrichment terms and downregulated hUMSC genes are included in the supplementary material 4 of sheet GO 4 and sheet 2.

### hAMSCs RNA-seq analysis

**Fig. 6:**
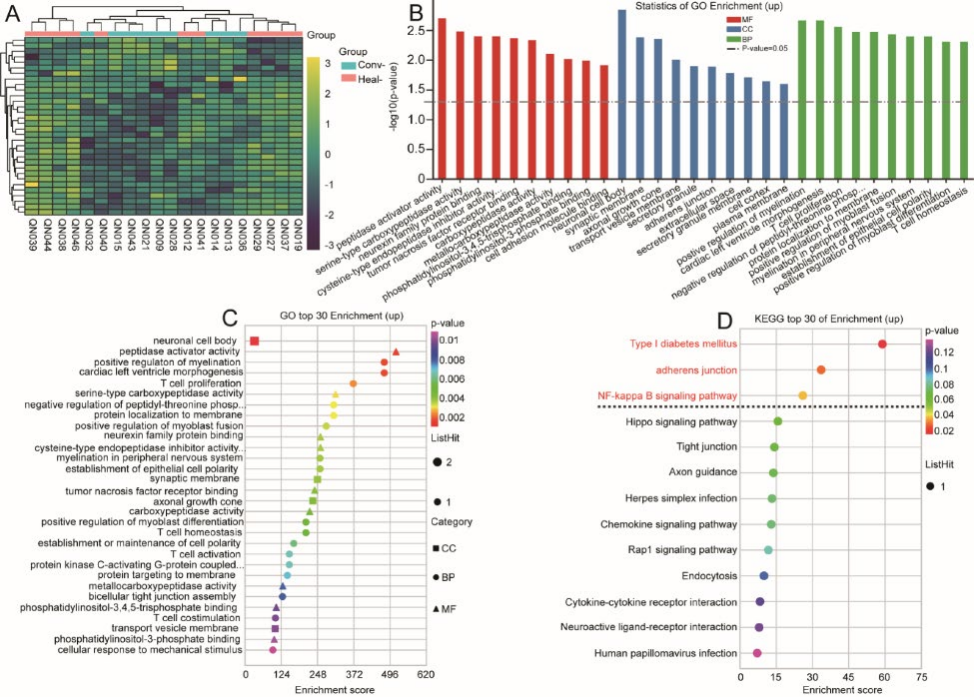
Differential expression of upregulated genes in hAMSCs between convalescent and control groups. **A:** Heatmap. Yellow and black stripes represent high and low gene expression, respectively. **B:** GO enrichment analysis. **C:** GO bubble chart. The vertical axis and horizontal axis represent the GO terms and enrichment score, respectively. **D:** KEGG bubble chart. The vertical axis and horizontal axis represent enrichment pathway and enrichment score, respectively. Conv-: convalescent COVID-19 pregnant women. Heal-: healthy pregnant women. MF: molecular function. CC: cellular component. BP: biological process

RNA-seq analysis was performed on hAMSCs from the convalescent and control groups. A total of 32 DEGs were identified of which 8 were upregulated genes and 24 were downregulated genes. Upregulated genes were used to perform GO enrichment and showed GO terms related to T cell proliferation (*p* = 0.0027), establishment of epithelial cell polarity (*p* = 0.0039), positive regulation of myoblast differentiation (*p* = 0.0048), T cell homeostasis (*p* = 0.0048), establishment or maintenance of cell polarity (*p* = 0.006), T cell activation (*p* = 0.0066), the protein kinase C-activating G-protein coupled receptor signaling pathway (*p* = 0.0066), bicellular tight junction assembly (*p* = 0.0078), T cell co-stimulation (*p* = 0.0096), cellular response to mechanical stimulus (*p* = 0.01), cell adhesion molecule binding (*p* = 0.012), the transforming growth factor beta receptor signaling pathway (*p* = 0.015), and the tumor necrosis factor-mediated signaling pathway (*p* = 0.021). KEGG pathway enrichment analysis of upregulated genes identified 10 pathways. Of these, 3 KEGG pathways were significantly enriched: type I diabetes mellitus (path: hsa04940, *p* = 0.017), adherens junction (path: hsa04520, *p* = 0.03), and the NF-kappa B signaling pathway (path: hsa04064, *p* = 0.038) (Fig. 6). The other GO enrichment terms, KEGG pathways, and upregulated hAMSC genes are included in the supplementary material 5 of sheet GO 5, sheet KEGG 4, and sheet 3.

**Fig. 7:**
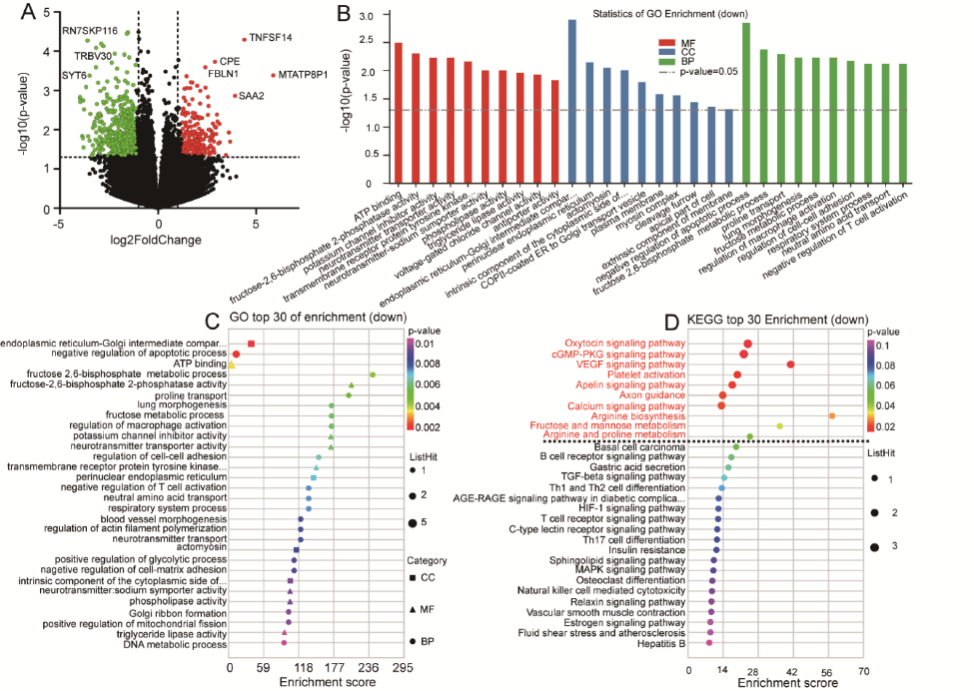
Differential expression of upregulated genes in hAMSCs between convalescent and control groups. **A:** Volcano plot. Red and green dots represent the upregulated and downregulated expressed genes respectively. **B:** GO enrichment analysis. **C:** GO bubble chart. The vertical axis and horizontal axis represent the GO terms and enrichment score, respectively. **D:** KEGG bubble chart. The vertical axis and horizontal axis represent enrichment pathway and enrichment score, respectively. MF: molecular function. CC: cellular component. BP: biological process

Downregulated genes were used to perform GO enrichment and showed GO terms related to negative regulation of the apoptotic process (*p* = 0.0014), regulation of macrophage activation (*p* = 0.0058), regulation of cell-cell adhesion (*p* = 0.0066), respiratory system processes (*p* = 0.0074), negative regulation of T cell activation (*p* = 0.0074), blood vessel morphogenesis (*p* = 0.0082), regulation of actin filament polymerization (*p* = 0.0082), negative regulation of cell-matrix adhesion (*p* = 0.009), lung alveolus development (*p* = 0.013), regulation of the MAPK cascade (*p* = 0.02), negative regulation of cell adhesion (*p* = 0.02), the epidermal growth factor receptor signaling pathway (*p* = 0.024), calcium-dependent protein binding (*p* = 0.037), the ephrin receptor signaling pathway (*p* = 0.042), and the transmembrane receptor protein tyrosine kinase signaling pathway (*p* = 0.048). KEGG pathway enrichment analysis of downregulated genes identified 24 pathways. Of these, 10 KEGG pathways were significantly enriched, including the oxytocin signaling pathway (path: hsa04921, *p* = 0.00017), the cGMP-PKG signaling pathway (path: hsa04022, *p* = 0.00021), the VEGF signaling pathway (path: hsa04370, *p* = 0.00096), platelet activation (path: hsa04611, *p* = 0.0041), the apelin signaling pathway (path: hsa04371, *p* = 0.0051), axon guidance (path: hsa04360, *p* = 0.0082), and the calcium signaling pathway (path: hsa04020, *p* = 0.0088). (Fig. 7). The other GO enrichment terms, KEGG pathways, and downregulated hAMSC genes are included in the supplementary material 6 of sheet GO 6, sheet KEGG 5, and sheet 3.

### ACE2 and TMPRSS2 expression

**Fig. 8:**
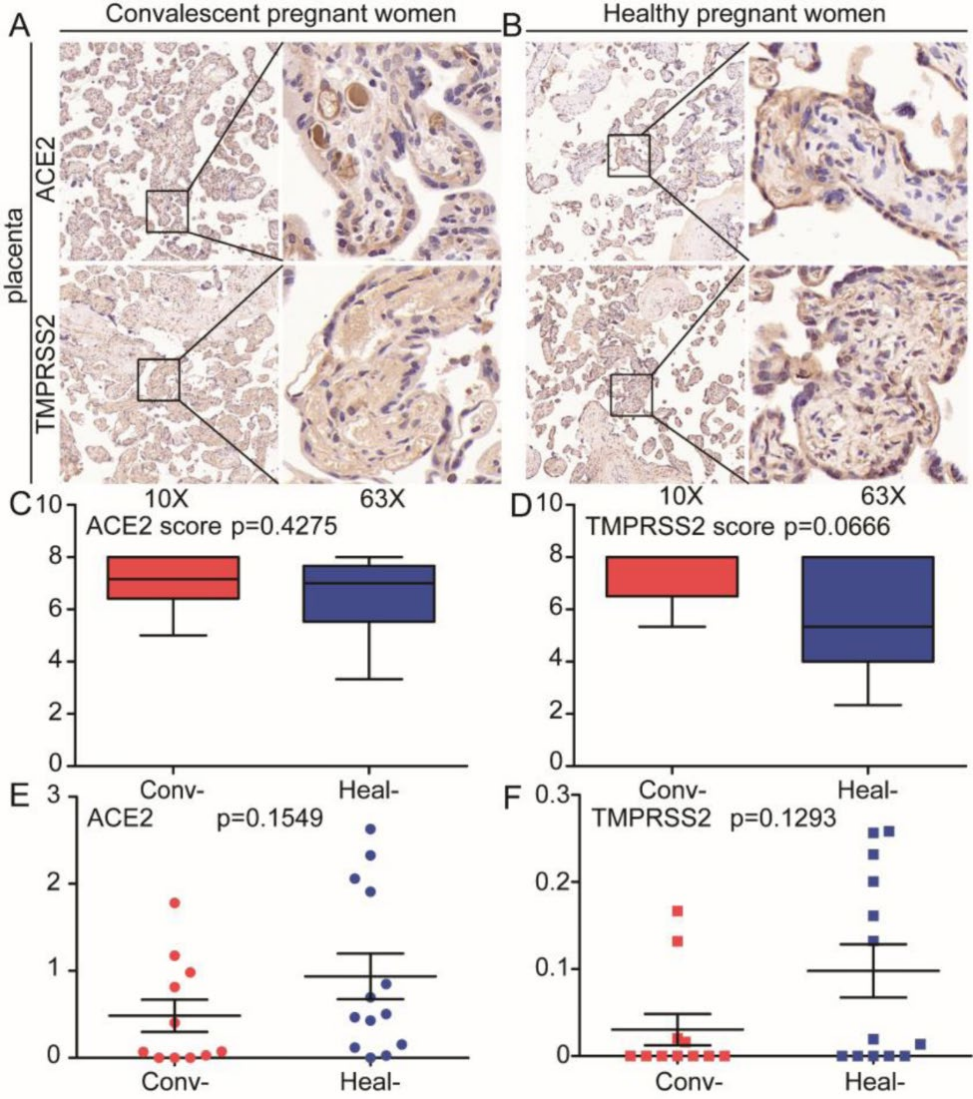
Immunohistochemical (IHC) staining for ACE2 and TMPRSS2 in placental tissue from convalescent and control groups. ACE2 and TMPRSS2 expression in placental tissue from convalescent **(A)** and control women **(B)**. IHC scores were calculated for placental staining of ACE2 **(C)** and TMPRSS2 expression **(D)**. RNA-seq analysis of placental tissue showed there was no difference in expression of ACE2 **(E)** and TPMRSS2 **(F)** between the two groups. Conv-: convalescent COVID-19 pregnant women. Heal-: healthy pregnant women

IHC staining of ACE2 and TMPRSS2 was performed on tissue specimens from the placenta in the convalescent and control groups. ACE2 and TMPRSS2 were both positive in the placenta from convalescent and control groups with no difference in the IHC scores between the two groups (*p* > 0.05). RNA-seq showed that there was no difference in the expression of these two biomarkers between the groups (*p* > 0.05) (Fig. 8).

### Cytokine measurements

**Fig. 9:**
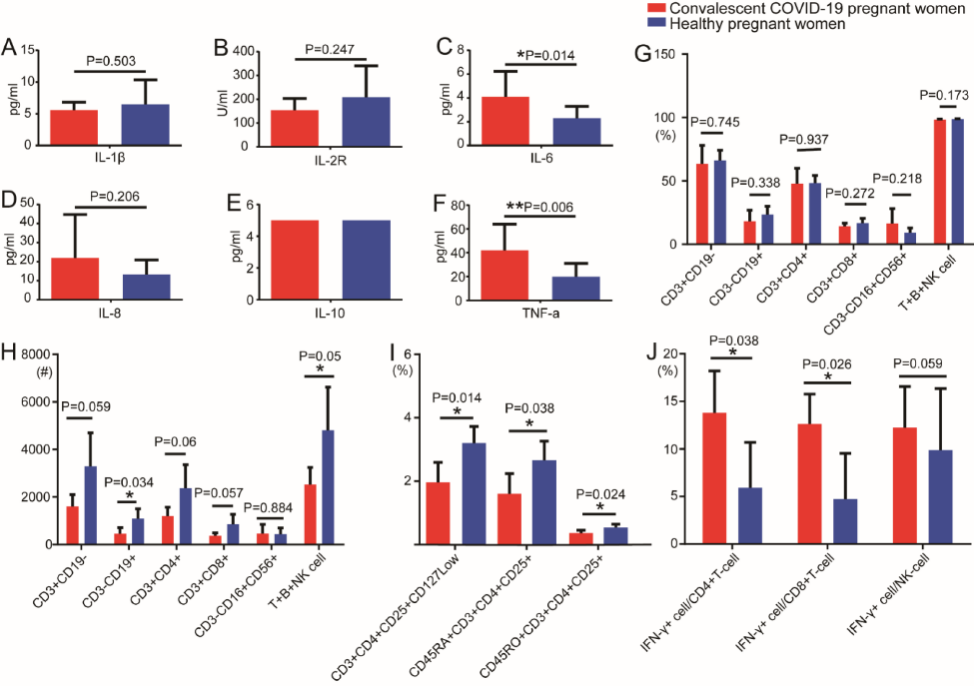
Umbilical cord blood cytokines, immune cell subsets, and function analysis in convalescent and control groups. A series of comparisons of plasma cytokine levels between convalescent (N = 9) and control women (N = 13) **(A-F)**. Flow cytometry staining of T cells, B cells, NK cells, Tregs, and IFN-γ expressed CD4^+^ T, CD8^+^ T, and NK cells. A series of comparisons of the proportion of total T cells, total B cells, CD4^+^ T cells, CD8^+^ T cells, NK cells, and total T + B + NK cells between convalescent (N = 4) and control women (N = 5) **(G)**. A series of comparisons of absolute number of total T cells, total B cells, CD4^+^ T cells, CD8^+^ T cells, NK cells, and total T + B + NK cells between convalescent (N = 4) and control women (N = 5) **(H)**. A series of comparisons of proportion of Tregs, CD45RA^+^ Tregs, and CD45RO^+^ Tregs between convalescent (N = 4) and control women (N = 5) **(I)**. A series of comparisons of the proportion of IFN-γ expressed by CD4^+^ T cells, CD8^+^ T cells and NK cells between convalescent (N = 4) and control women (N = 5) **(J)**. All data are presented as mean ± SEM.

The plasma from UCB of 9 convalescent women and 13 control women was used to perform cytokine tests. The results are shown in Fig. 9. The detailed statistics of the differences in cytokine expression between the two groups are shown in Table S1. IL-6 and TNF-α were statistically different between the convalescent and control groups (4.1 ± 2.1 versus 2.3 ± 1.0, *p* = 0.014; 42.0 ± 22.0 versus 19.9 ± 11.1, *p* = 0.006, respectively).

### Umbilical cord blood immunological indicators

UCB cells from 4 convalescent women and 5 control women were used to perform the analysis of lymphocyte subsets and functional analyses. The results are shown in Fig. 9. The plasma IL-6 and TNF-α levels, but not IL-1β, IL-2R, IL-8, and IL-10 levels, were statistically significant higher in the convalescent women than the control women (Fig. 9A-F). Although there was no difference in the percentage of immune cells subsets (T lymphocytes, B lymphocytes, helper/induced T Lymphocytes, inhibitory/cytotoxic T lymphocytes, NK cells, and total lymphocytes) (Fig. 9G), the absolute number of B lymphocytes and total lymphocytes (T + B + NK cells) were statistically different between the convalescent and control groups (455.8 ± 255.5 versus 1087.2 ± 419.1, *p* = 0.034; 2523.5 ± 718.5 versus 4809.8 ± 1797.0, *p* = 0.050, respectively) (Fig. 9H). The percentage of regulatory T cells (CD3^+^CD4^+^CD25^+^CD127^Low^), natural regulatory T cells (CD45RA^+^CD3^+^CD4^+^CD25^+^), and induced regulatory T cells (CD45RO^+^CD3^+^CD4^+^CD25^+^) were statistically different between the convalescent and control groups (2.0 ± 0.6 versus 3.2 ± 0.5, *p* = 0.014; 1.6 ± 0.6 versus 2.7 ± 0.6, *p* = 0.038; 0.4 ± 0.1 versus 0.5 ± 0.1, *p* = 0.024, respectively) (Fig. 9I). IFN-γ expressed CD4^+^ T and CD8^+^ T cells were statistically different between the convalescent and control groups (13.8 ± 4.4 versus 5.9 ± 4.8, *p* = 0.038 and 12.6 ± 3.1 versus 4.7 ± 4.8, *p* = 0.026, respectively) (Fig. 9J). The detailed statistical analysis of the two groups and different immune cell subsets and functional analyses are shown in Table S2.

## Discussion

The specific effects of SARS-CoV-2 on pregnancy and fetal development remain largely unknown [13, 28–30]. Of note, the majority of neonates born to COVID-19 positive pregnant women were not also infected [8–12]. In addition, no SARS-CoV-2 transcript was detected in the placenta cells, hUMSCs, and hAMSCs. In this study, we performed RNA-seq on placental tissue and analyzed the DEGs between pregnant women who had recovered from COVID-19 and healthy pregnant women. GO and KEGG analysis showed that these DEGs were associated with cell proliferation, cell-cell adhesion, cell-cell adherens junctions, inflammatory responses, and immune responses. EIF5B, one of the important DEGs, is involved in RNA transport pathway (path:hsa03013). EIF5B is an initiation factor to regulate the initiation of translation along with cells proliferation, inhibition of apoptosis, and immunosuppression under stress conditions. EIF5B is reported to act on the internal ribosome entry site to mediate the translation of several viral and anti-apoptotic mRNA [31]. Lu-Culligan et al. used bulk-RNA sequencing of placental villi to examine differences in the placental gene expression between COVID-19 pregnant women and uninfected healthy pregnant women. Their results showed increased expression of genes associated with the immune response. The most significantly upregulated gene was HSPA1A. In the single cell transcriptomic profiling of the placenta, the placental NK cells had significantly enriched genes encoding cytotoxic proteins, including GZMA, GZMB, and GNLY, and tissue-repair growth factor AREG, during the COVID-19 infection. T cell subsets upregulated CD69 and encoded ribosomal proteins RPL36 and RPS10. In addition, endothelial cells upregulated the interferon-reduced protein ISG15 and critical regulators of the NF-KB pathway NFKBIA and NFKBIZ [32]. We speculated that the placenta acts as a barrier and plays a vital role in blocking the vertical transmission of the virus between pregnant women and their fetuses.

In the present study, there were differences in cytokine levels as well as lymphocyte subpopulations between the convalescent women and healthy controls. IL-6 and TNF-α were increased and immune cell counts were decreased in UCB from convalescent women compared with healthy controls. Fernandes et al. demonstrated significantly higher levels of IL-6, TNF-α, and IFN-γ in convalescent COVID-19 pregnant women than healthy pregnant women in matched trimesters [33]. Zhao et al. reported that most of the immune cell subsets in the peripheral blood returned to the normal levels with no significant changes in UCB in convalescent COVID-19 pregnant women [34]. Kuri-Cervantes et al. showed that the proportions of T cells, CD8 + MAIT cells, ILCs, and NK cells in convalescent COVID-19 pregnant women were similar to those of healthy pregnant women [35]. These results were consistent with those seen in COVID-19 patients in the early stages of infection rather than serious second stage characterized by extensive inflammation [36]. The reduction of T-regs and effector T cells maintained the immune response against SARS-CoV-2 [37]. Those changes might be one of the most important protective mechanisms for the fetus [4, 38]. An increased number of IFN-γ expressing CD4^+^ T cells, CD8^+^ T cells, and NK cells were found in UCB from convalescent pregnant women, in levels similar to the peripheral blood levels of these immune cells seen in non-pregnant patients with mild and moderate COVID-19 [39]. Chen et al. performed the single-cell mRNA sequencing and single-cell TCR sequencing in the peripheral blood mononuclear cells isolated from the convalescent pregnant women and healthy controls. They found normal clonal expansion of T cells, heightened activation and chemotaxis in NK, NKT, and MAIT cells, and differential interferon responses in the monocyte compartment [40]. Immune cells of UCB are in a state of dynamic equilibrium, thus the functions of hUMSCs and hAMSCs may undergo changes in pregnant women who have recovered from COVID-19 infection. As MSCs are immunomodulatory and have important roles in maintaining immune balance, we also speculate that MSCs play an important role in maintaining fetal and maternal immune responses.

Some patients display persistent symptoms after acute COVID-19 infection. Comparing the differences in the gene expression of hUMSCs and hAMSCs derived from convalescent pregnant women and health pregnant women using RNA-Seq, we found some DEGs between these two groups. Enrichment analysis showed those DEGs were associated with immune homeostasis, antiviral activity, cell proliferation, and tissue repair. PIPOX, as one of the important DEGs in hUMSCs, is involved in Glycine, serine, and threonine metabolism pathway (path:hsa00260). PIPOX is involved in the pipecolate protection, which influences cell signaling during oxidative stress to promote cell survival, localize to mitochondria, with a cellular stress protection mechanism similar to that of proline [41]. TNFSF14, as another important DEGs in hAMSCs, is involved in the NF-kappa B signaling pathway (path:hsa04064). TNFSF14 is an important modulator of critical innate and adaptive immune responses and plays an important role in the COVID-19 pathogenesis [42]. TNFSF14 creates a self-regulating host defense system, which plays a key role in communication to control the immune response [43]. Therefore, MSCs could have a great potential in the treatment of COVID-19 patients with persistent symptoms during recovery stage.

The limitations of our study include its small sample size and recruitment of participants from a single research center. We did not consider the impact of delivery modes on the test and sequencing results. Different SARS-CoV-2 variants may also cause different immune responses. In addition, we performed a cross-sectional study and were not able to establish a causal relationship between these inflammatory changes and clinical manifestations. The changes in MSCs were only detected by transcriptome level and experiments to analyze proteomic and metabolomic changes were not performed. However, we did provide a comprehensive analysis of the inflammatory changes in pregnant women who had recovered COVID-19 and their fetuses, which could provide a framework for future research.

## Conclusion

We identified multiple immune responses in convalescent women and their fetuses after acute COVID-19 infection. Some of these immune responses may suggest that hUMSCs and hAMSCs derived from pregnant women after recovery could have therapeutic effects in the treatment of COVID-19.

### Electronic supplementary material

Below is the link to the electronic supplementary material.

Supplementary Material 1

Supplementary Material 2

Supplementary Material 3

Supplementary Material 4

Supplementary Material 5

Supplementary Material 6

Supplementary Material 7

Supplementary Material 8

Supplementary Material 9

Supplementary Material 10

Supplementary Material 11

## Data Availability

The datasets generated and/or analysed during the current study are available in the [BioProject] repository, [http://www.ncbi.nlm.nih.gov/bioproject/912358; ID PRJNA912358].

## List of abbreviations

ACE2: Angiotensin converting enzyme-2
COVID-19: Coronavirus disease 2019
DEGs: Differentially expressed genes
GO: Gene ontology
hAMSCs: Human amniotic mesenchymal stem cells
hUMSCs: Human umbilical cord mesenchymal stem cells
IHC: Immunohistochemical staining
KEGG: Kyoto encyclopedia of genes and genomes
PCR: Polymerase chain reaction
UCB: Umbilical cord blood
TMPRSS2: Transmembrane serine protease 2
