# Supplementary material for: Immune and cytokine alterations and RNA-sequencing analysis in gestational tissues from pregnant women after recovery from COVID-19
Source: BMC Infect Dis. 2023 Sep 21;23:620. doi: 10.1186/s12879-023-08607-z (PMC10512579; doi:10.1186/s12879-023-08607-z)
Supplement: Supplementary file 7 — Supplementary Material 7 [file 12879_2023_8607_MOESM7_ESM.docx]

IHC staining score protocol and rules:

Six researchers scored the IHC staining, and the final results were averaged. IHC scoring rules are detailed in the supplementary materials. Immunohistochemical staining results were assigned a mean score considering both the intensity of staining and the proportion of cells with positive reaction. The intensity was scored as follows: 0, negative; 1, weak; 2, moderate; and 3, strong. The frequency of positive cells was defined as follow: 0, less than 5%; 1, 5% to 25%; 2, 26% to 50%; 3, 51% to 75%; and 4, great than 75%. IHC staining scores was determined by multiplying the score for staining intensity with the score for positive area. Scores of 0 to 7 were considered low expression and scores of 8 to 12 considered high expression.
