# Supplementary material for: Immune and cytokine alterations and RNA-sequencing analysis in gestational tissues from pregnant women after recovery from COVID-19
Source: BMC Infect Dis. 2023 Sep 21;23:620. doi: 10.1186/s12879-023-08607-z (PMC10512579; doi:10.1186/s12879-023-08607-z)
Supplement: Supplementary file 8 — Supplementary Material 8 [file 12879_2023_8607_MOESM8_ESM.docx]

Table S1. The detection value of umbilical cord blood of cytokine in different specimens.

| Patients ID. | P/N | Specimen ID. | IL-1B | IL-2R | IL-6 | IL-8 | IL-10 | TNFa |
| --- | --- | --- | --- | --- | --- | --- | --- | --- |
| C3 | P | HSC002 | 5 | 115 | 7.17 | 19.6 | 5 | 23.7 |
| C4 | P | HSC005 | 5.8 | 172 | 6.32 | 16.8 | 5 | 75.7 |
| C5 | P | HSC008 | 8.7 | 157 | 4.45 | 17.6 | 5 | 72 |
| CN9 | N | HSC011 | 5 | 183 | 1.69 | 16.8 | 5 | 45.6 |
| C6 | P | HSC012 | 5 | 151 | 2.19 | 8.4 | 5 | 39.1 |
| C7 | P | HSC013 | 5 | 164 | 2.7 | 81.7 | 5 | 26.4 |
| C8 | P | HSC014 | 5 | 83 | 1.98 | 14.7 | 5 | 40.6 |
| CN12 | N | HSC016 | 5 | 170 | 1.5 | 7.1 | 5 | 12.6 |
| CN13 | N | HSC020 | 5 | 186 | 2.06 | 12.1 | 5 | 33.8 |
| CN14 | N | HSC021 | 5 | 338 | 1.77 | 6.8 | 5 | 11.5 |
| C11 | P | HSC024 | 6 | 105 | 1.94 | 17.8 | 5 | 55.2 |
| CN15 | 2 | HSC027 | 5 | 336 | 2.43 | 8.5 | 5 | 9.8 |
| C12 | P | HSC028 | 5 | 237 | 6.65 | 13.5 | 5 | 10.2 |
| CN16 | N | HSC029 | 5.1 | 507 | 5.37 | 5.4 | 5 | 17.1 |
| CN17 | N | HSC030 | 5 | 150 | 1.67 | 8.9 | 5 | 33.9 |
| CN19 | N | HSC031 | 17.3 | 50 | 2.33 | 13.4 | 5 | 11.3 |
| CN18 | N | HSC032 | 5 | 319 | 2.43 | 6.3 | 5 | 14.8 |
| CN20 | N | HSC033 | 5 | 146 | 2.05 | 15 | 5 | 19.7 |
| C13 | P | HSC034 | 5 | 208 | 3.59 | 7.8 | 5 | 34.8 |
| CN21 | N | HSC035 | 5 | 182 | 2.38 | 25.7 | 5 | 14.9 |
| CN22 | N | HSC036 | 5 | 106 | 2.72 | 14.6 | 5 | 22 |
| CN23 | N | HSC037 | 12.3 | 50 | 1.51 | 30.6 | 5 | 12.1 |

P: positive (convalescent COVID-19 pregnant women); N: negative (healthy control pregnant women)
