# Supplementary material for: Immune and cytokine alterations and RNA-sequencing analysis in gestational tissues from pregnant women after recovery from COVID-19
Source: BMC Infect Dis. 2023 Sep 21;23:620. doi: 10.1186/s12879-023-08607-z (PMC10512579; doi:10.1186/s12879-023-08607-z)
Supplement: Supplementary file 9 — Supplementary Material 9 [file 12879_2023_8607_MOESM9_ESM.docx]

Table S2. The detailed statistics of two groups and different immune cell subsets and function analysis

| Patients ID. | C5 | CN8 | CN9 | C6 | C7 | C8 | CN12 | CN13 | CN14 |
| --- | --- | --- | --- | --- | --- | --- | --- | --- | --- |
| P/N | P | N | N | P | P | P | N | N | N |
| Specimen ID. | HSC008 | HSC010 | HSC011 | HSC012 | HSC013 | HSC014 | HSC016 | HSC020 | HSC021 |
| (CD3+CD19-)% | 48.59 | 79.17 | 64.25 | 56.93 | 82.34 | 66.52 | 57.01 | 66.62 | 63.73 |
| (CD3+CD19-)# | 1559 | 4589 | 4034 | 1608 | 2231 | 1007 | 1423 | 4257 | 2138 |
| (CD3-CD19+)% | 25.81 | 15.11 | 28.35 | 12.81 | 9.1 | 25.48 | 30.94 | 18.53 | 24.58 |
| (CD3-CD19+)# | 828 | 875 | 1780 | 362 | 247 | 386 | 772 | 1184 | 825 |
| (CD3+CD4+)% | 34.47 | 58.73 | 44.41 | 42.01 | 62.47 | 52.39 | 45.24 | 47.4 | 45.91 |
| (CD3+CD4+)# | 1106 | 3404 | 2788 | 1187 | 1693 | 793 | 1129 | 3028 | 1540 |
| (CD3+CD8+)% | 12.67 | 19.39 | 18.81 | 13.46 | 17.89 | 13.02 | 10.48 | 17.84 | 17.43 |
| (CD3+CD8+)# | 406 | 1124 | 1181 | 380 | 485 | 197 | 262 | 1140 | 585 |
| (CD3-/CD16+CD56+)% | 24.27 | 4.31 | 6.78 | 28.67 | 6.4 | 6.85 | 10.94 | 13.83 | 10.11 |
| (CD3-/CD16+CD56+)# | 779 | 250 | 426 | 810 | 173 | 104 | 273 | 884 | 339 |
| T+B+NK% | 98.67 | 98.59 | 99.38 | 98.41 | 97.84 | 98.85 | 98.89 | 98.98 | 98.42 |
| T+B+NK# | 3166 | 5714 | 6240 | 2780 | 2651 | 1497 | 2468 | 6325 | 3302 |
| CD3+CD4+CD25+CD127Low | 1.53 | 3.75 | 2.64 | 1.44 | 2.03 | 2.82 | 3.01 | 2.85 | 3.75 |
| CD45RA+CD3+CD4+CD25+ | 1.13 | 3.27 | 1.99 | 1.04 | 1.8 | 2.42 | 2.52 | 2.21 | 3.32 |
| CD45RO+CD3+CD4+CD25+ | 0.4 | 0.48 | 0.65 | 0.4 | 0.23 | 0.4 | 0.49 | 0.64 | 0.43 |
| IFN-γ+/CD4+ T cells | 11.11 | 1.08 | 2.79 | 18.58 | 16.32 | 9.21 | 3.73 | 11.87 | 10.11 |
| IFN-γ+/CD8+ T cells | 16.08 | 3.14 | 1.11 | 14.24 | 11.09 | 9.08 | 3.08 | 13.19 | 3.17 |
| IFN-γ+/NK cells | 12.55 | 6.76 | 4.56 | 13.15 | 16.83 | 6.38 | 5.98 | 11.86 | 20.35 |

P: positive (convalescent COVID-19 pregnant women); N: negative (healthy control pregnant women).
