# Supplementary material for: Immune and cytokine alterations and RNA-sequencing analysis in gestational tissues from pregnant women after recovery from COVID-19
Source: BMC Infect Dis. 2023 Sep 21;23:620. doi: 10.1186/s12879-023-08607-z (PMC10512579; doi:10.1186/s12879-023-08607-z)
Supplement: Supplementary file 10 — Supplementary Material 10 [file 12879_2023_8607_MOESM10_ESM.docx]

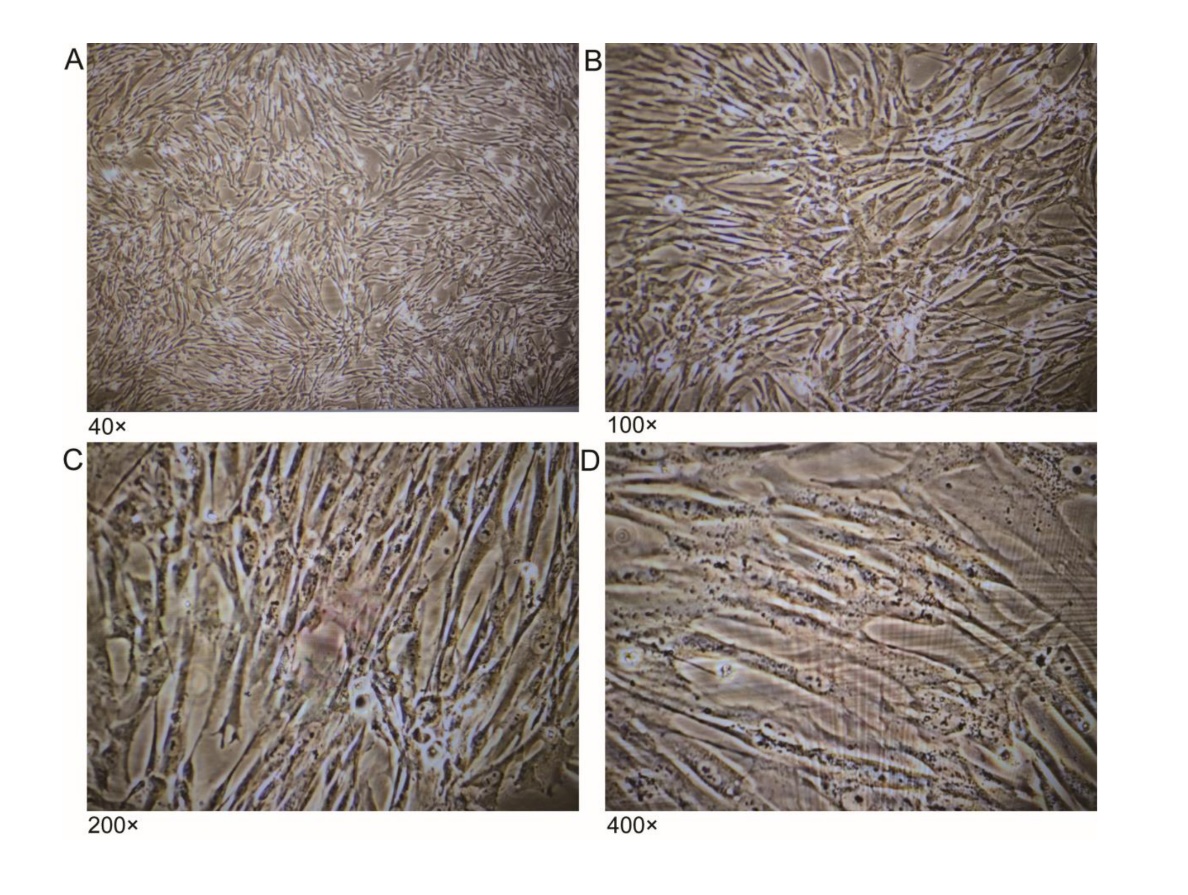


Supplemental Material Figure S1. Microscopy images of hUMSCs (A, B, C, and D show different magnification levels).
